# dBcAMP Rescues the Neurons From Degeneration in Kainic Acid-Injured Hippocampus, Enhances Neurogenesis, Learning, and Memory

**DOI:** 10.3389/fnbeh.2020.00018

**Published:** 2020-03-05

**Authors:** Muddanna Sakkattu Rao, Ebtesam M. Abd-El-Basset

**Affiliations:** Department of Anatomy, Faculty of Medicine, Kuwait University, Kuwait, Kuwait

**Keywords:** dBcAMP, doublecortin, hippocampus, Morris water maze, hippocampal neurogenesis, passive avoidance

## Abstract

Dibutyryl cyclic adenosine monophosphate (dBcAMP) is a cell-permeable synthetic analog of cyclic adenosine monophosphate (cAMP). Although the elevation of cAMP levels was reported to promote the functional recovery in spinal cord injury, its role in neurogenesis or functional recovery after hippocampal injury is unknown. The objective of the study was to investigate the effects of dBcAMP on learning, memory, and hippocampal neurogenesis in the excitotoxically lesioned hippocampus. An excitotoxic lesion was induced in the hippocampi of 4-month-old male BALB/c mice by injecting 0.25 μg/μl into the lateral ventricles of both sides. The lesioned mice (L) were divided into L+dBcAMP and L+phosphate-buffered saline (PBS) groups. Sham surgery (S) was done by the injection of 1 μl of sterile saline into the lateral ventricles. The sham surgery mice were divided into S+dBcAMP and S+PBS groups. Mice in the L+dBcAMP and S+dBcAMP groups were treated with dBcAMP for 1 week (i.p., 50 mg/kg), whereas mice in the L+PBS and S+PBS groups were treated with PBS. The mice in all groups were subjected to water maze and passive avoidance tests at the end of the 4th week. Cresyl violet staining and NeuN and doublecortin immunostaining were done to analyze the morphology and neurogenesis. The water maze learning sessions did not show a significant difference in escape latency between the groups, suggesting an unimpaired learning ability of mice in all groups. The L+dBcAMP mice had significantly short entry latency and higher target quadrant time/distance traveled compared to the L+PBS group, suggesting better memory retention. The L+dBcAMP group had a significantly improved memory retention compared to the L+PBS mice during the passive avoidance test. Morphological studies showed significantly greater adult neurons and increased hippocampal neurogenesis in the hippocampus of mice in the L+dBcAMP group compared to those in the L+PBS group. There was no significant difference between the S+dBcAMP and S+PBS groups in the water maze/passive avoidance tests and the number of neurons. In conclusion, dBcAMP protects the hippocampal neuron from degeneration and enhances hippocampal neurogenesis, learning, and memory.

## Introduction

In young individuals, traumatic brain injury is a known cause of morbidity and mortality. Traumatic forces during injury produce immediate neurologic damage. Several neural (neurons, axons, and dendrites) and non-neural structures (glia and blood vessels) are damaged by traumatic forces directly in a focal, multifocal, or diffuse pattern (Mckee and Daneshvar, 2015). A set of secondary metabolic and cellular changes is triggered by primary injury. Neuronal death after acute brain injury occurs initially by necrosis, and apoptosis is mostly the path of delayed cell death (Zhang et al., 2005; Krishnamurthy and Laskowitz, 2016; McDonald et al., 2016; Plummer et al., 2016).

Brain injury is known to stimulate neurogenesis. Adult neurogenesis is a process of addition of new neurons in the adult brain. In adult neurogenesis, the neural progenitor cells proliferate and differentiate into mature neuronal phenotypes. Neurogenesis occurs in the sub-granular zone (SGZ) of the dentate gyrus (DG) of the hippocampus and in the sub-ventricular zone (SVZ) of the lateral ventricles (Gage, 2002; Taupin and Gage, 2002). The significance of the injury that stimulated adult neurogenesis may be to replace the lost neurons in both the SVZ and the DG regions (Emery et al., 2005; Rola et al., 2006; Kernie and Parent, 2009; Blaiss et al., 2011). Neurons born after the injury contribute to post-injury structural and functional recovery (Emery et al., 2005; Kernie and Parent, 2009; Blaiss et al., 2011). Adult neurogenesis from the proliferating stem/progenitor cells in the SGZ of the DG is known in many animal species, including humans (Kuhn et al., 1996; Cameron and McKay, 1998; Eriksson et al., 1998; Kornack and Rakic, 1999; Gould and Gross, 2002; van Praag et al., 2002). Adult neurogenesis in the DG is related with learning and memory functions of the hippocampus (Gross, 2000; Shors, 2001; Monje et al., 2003). The rate of stem cell proliferation and the extent of differentiation of progenitor cells into the neurons in the hippocampal DG may be altered by changes in the stem/progenitor cell environment in the SGZ (Monje et al., 2002; Monje and Palmer, 2003). Ischemia, stroke, and hypoxia are reported to enhance dentate neurogenesis (Felling and Levison, 2003). Status epilepticus, induced by several standard methods including intracerebro-ventricular kainic acid infusion, is shown to increase cell division and neurogenesis in the SGZ of the DG in the acute phase of epileptogenesis (during the first week after the injury; Parent et al., 1997; Gray and Sundstrom, 1998; Madsen et al., 2000; Nakagawa and Yuan, 2000). However, adult dentate neurogenesis returns to a normal level a few weeks after the injury (Parent et al., 1997; Nakagawa and Yuan, 2000). In the case of the chronic phase of temporal lobe epilepsy, neurogenesis drastically decreases (Hattiangady et al., 2004). Hippocampal injury can lead to decreased dentate neurogenesis, which in turn can lead to learning and memory deficit (Zhang et al., 2015; Lee et al., 2017; Sakhaie et al., 2017). Decreased hippocampal-dependent learning and memory functions and decreased neurogenesis are associated with each other (Mikati et al., 2001; Alessio et al., 2004).

Therefore, strategies that enhance the potential of the brain have significant roles in functional recovery after neural injury. Neurotrophic factors, such as brain-derived neurotrophic factor (BDNF), basic fibroblast growth factor (bFGF), and vascular endothelial growth factor (VEGF), are known to stimulate adult neurogenesis (Cao et al., 2004; Kang and Hébert, 2015; Wei et al., 2015; Han et al., 2017; Numakawa et al., 2018). In addition, other factors such as dibutyryl cyclic adenosine monophosphate (dBcAMP), an analog of cyclic adenosine monophosphate (cAMP), have been reported to promote functional recovery in the spinal cord injury (Myeku et al., 2012; Xia et al., 2013). However, its role in hippocampal injury is unknown. In an astroglia culture, the dBcAMP is known to induce astrogliosis (Fedoroff et al., 1984; Abd-El-Basset, 2000). Following the traumatic neural injury, synthesis of many cytokines, growth factors, and neuropeptides occurs in reactive astrocytes. This suggests that the astrocytic reaction may play an important role in neuronal regeneration (Faulkner et al., 2004; John et al., 2005; Rolls et al., 2009; Colangelo et al., 2014; Pekny et al., 2014). Astrocytes play a role in the regulation of neurogenesis, the integrity of the blood–brain barrier, and the neuronal metabolic homeostasis (Castronguay and Robitaille, 2001; Fields and Stevens-Graham, 2002; Garner et al., 2002). Our recent study had shown a neuroprotective role of dBcAMP in a stab wound and intracerebro-ventricular kainic acid model of brain injury by enhancing the astrocytes and the microglia in the region of the stab wound and the kainic acid-induced hippocampal lesion (Abd-El-Basset and Rao, 2018). In addition, dBcAMP was found to enhance the BDNF level in the tissue around the injury site and in the hippocampus. The question on whether dBcAMP induced neuroprotection in the hippocampus, which leads to an enhancement of hippocampal neurogenesis and hence of learning, memory, and functional recovery, has not been addressed. Accordingly, the objective of the present study is to investigate the effects of dBcAMP on neurogenesis, learning, and memory in mice with excitotoxic hippocampal injury. We used the Morris water maze and the passive avoidance test to assess learning and memory at 4 weeks after the excitotoxic hippocampal injury. Neurogenesis was assessed by labeling the newly born neurons with doublecortin (DCX), a marker for newly born, young neurons.

## Materials and Methods

### Animals

Male Balb/c mice were used in the present study. The mice were maintained in the central animal research facility of the Faculty of Medicine, Kuwait University. The mice were fed with food and water *ad libitum*. The animals were maintained in 12:12 h dark/light cycle, and the room condition was maintained at a constant temperature (25 ± 2°C) and relative humidity (50 ± 10%). Animal care was observed according to the recommendations of the NIH Guidelines and the Guide for the Care and Use of Laboratory animals (Kuwait University—Faculty of Health Publication). All efforts were made to minimize the number of animals used and their suffering.

### Experimental Design

An excitotoxic lesion was created in the hippocampi of 4-months-old male BALB/c mice by injecting 1 μl (0.25 μg) of kainic acid (Sigma–Aldrich, St. Louis, MO, USA) into the lateral ventricle of both sides. Lesioned mice (L) were divided into L+dBcAMP and L+phosphate-buffered saline (PBS) groups. Sham surgery (S) was done by the injection of 1 μl of sterile saline into the lateral ventricles of both sides. The sham surgery mice were divided into S+dBcAMP and S+PBS groups. The mice in the L+dBcAMP and S+dBcAMP groups were treated with dBcAMP (Sigma–Aldrich, St. Louis, MO, USA) daily through the intraperitoneal route at a dose of 50 mg/kg for 1 week (i.p., 50 mg/kg/day) from the day of lesion, whereas the mice in the L+PBS and S+PBS groups were treated with PBS. A total of 48 mice (*n* = 12/group) were used in the study (Table 1). The mice in all groups were subjected to the water maze and the passive avoidance tests at the end of the 4th week. Morphological studies using Cresyl violet staining, NeuN immunostaining (for neuron), and DCX immunostaining (for neurogenesis) of brain sections were done. Hippocampal tissues were further analyzed for doublecortin content by Western blot.

**Table 1:** The number of mice, in different groups, which were used for the morphological and biochemical studies.

Groups	Morphological studies (doublecortin and NeuN immunostaining, Cresyl violet staining)	Biochemical studies (Western blot analysis for doublecortin)	Total (used for behavioral tests)
S+PBS	6	6	12
S+dBcAMP	6	6	12
L+PBS	6	6	12
L+dBcAMP	6	6	12
		Total	48

## Excitotoxic Lesion (Intracerebro-Ventricular Kainic Acid) Model of Brain Injury

In this experiment, an excitotoxic lesion (intracerebro-ventricular kainic acid, ICV-KA) model of brain injury/lesion was used. A lesion was made in both the left and the right hippocampi of 4-month-old BALB/c mice by injecting 1 μl (0.25 μg) of kainic acid (Sigma–Aldrich, St. Louis, MO, USA) into the lateral ventricle. The mice were anesthetized with a cocktail of ketamine (40 mg/kg) and xylocane (5 mg/kg). The mouse was held in the stereotaxic apparatus in such a way that the skull surface was horizontal in position. The skull surface was exposed by a midline skin incision. A burr hole was drilled with a dental drill [1.5 mm posterior to the bregma and 3 mm to the right lateral side and the left lateral side of the midline, following Paxinos and Watson mouse brain atlas (3rd edition)]. One microliter of kainic acid (0.25 μg/μl sterile saline; Sigma Chemicals, St. Louis, MO, USA) was injected into the lateral ventricles with a Hamilton syringe fitted with a 26G needle, which was inserted through the burr hole to a depth of 3.5 mm from the skull surface. KA injection was done slowly over a period of 10 min (0.1 μl/min). The needle was held in the same position for 20 min before withdrawing it to prevent a backflow of the injected KA. The skin was sutured, and antiseptic betadine solution was applied on the wound. The analgesic agent xylazine (2%) was injected (0.2 ml) around the suture every 8 h for 24 h for peri-operative pain relief. In sham surgery mice, 1 μl of sterile saline was injected into the lateral ventricles using the same surgery protocol and stereotaxic coordinates. The mice were kept under post-operative care in sterile cages. The first dose of dBcAMP (i.p., 50 mg/kg) or PBS was given 1 h after the KA injection and was continued for 1 week. The mice in all groups were maintained for 4 weeks.

### Spatial Learning and Memory Testing (Morris Water Maze Test)

**Figure 1:**
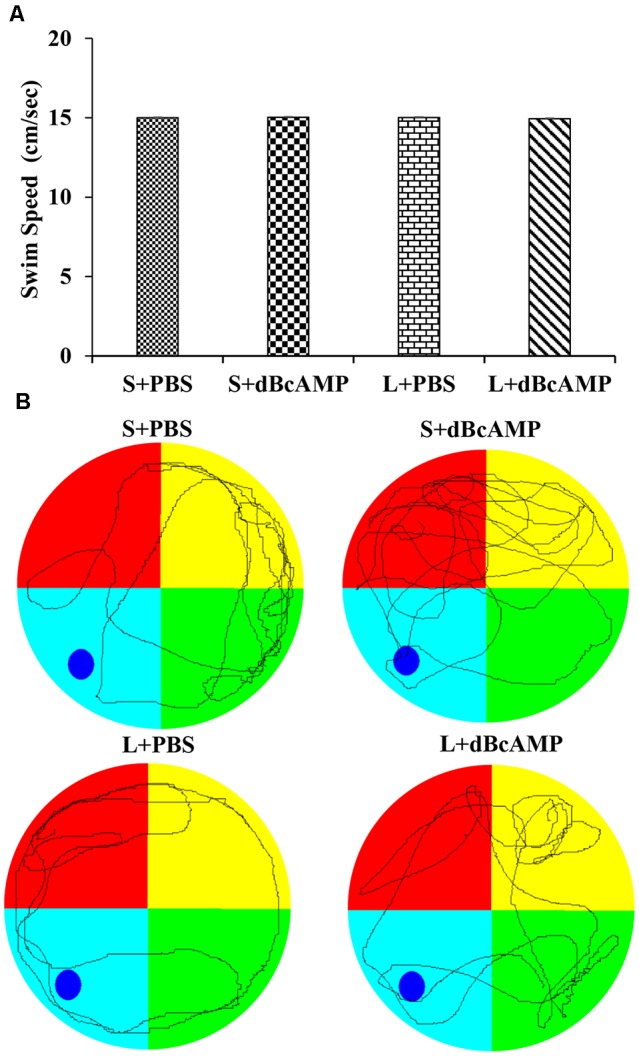
**(A)** Graph showing the swim speed of mice in the different groups during the screening test before the water maze learning sessions. Note that there is no significant difference between any groups (two-way ANOVA, *P* > 0.05; main interaction df-1, *F*_(1,44)_ = 0.02908, *P* = 0.8654; lesion type (L or S) df-1, *F*_(1,44)_ = 0.01663, *P* = 0.8980; treatment type (dBcAMP or PBS) df-1, *F*_(1,44)_ = 0.006245, *P* = 0.9374). **(B)** Representative video tracking of mice in the different groups during the screening test.

At the end of the experiment (4 weeks), the mice in all groups were tested in Morris water maze for spatial learning and memory (*n* = 12/group). Before subjecting the mice to the water maze test, all mice were screened for their swimming ability. The mice were allowed to swim in the water maze tank (100 cm in diameter) for 90 s in each trial, and the distance traveled was measured with an EzVideoTM 5.70 Digital Video Tracking System. For each mouse, three trials were given at an inter-trial interval of 2 min. The swim speed (cm/s; distance traveled in centimeter/90 s) was calculated for each mouse. In all groups, only the mice exhibiting a swim speed of 13–17 cm/s were used for the behavioral study (Figure 1). The water maze consisted of a water tank (1.0 m in diameter). The area of the tank water surface was divided into four virtual quadrants. The depth of the water tank was 20 cm. In the target/platform quadrant, a circular platform was submerged 1.0 cm below the water level. The mice were trained in the water maze on five consecutive days (a total of nine sessions: one session on the first day and two sessions each on the second to the 5th day). Each session consisted of four trials; each trial was of 90-s duration. The inter-trial interval was 60 s. In each trial, the time taken to reach the hidden platform (escape latency) was measured and analyzed using an EzVideo^TM^ 5.70 Digital Video Tracking System (Accuscan Instruments, Inc., Columbus, OH, USA). At 24 h after the last learning session, the mice were subjected to a memory retention test. Each memory retention test (probe test) session was of 30 s in duration. During the probe/memory retention test, the platform quadrant entry latency, the distance traveled in the target/platform quadrant, and the time spent in the target quadrant were measured and analyzed using an EzVideoTM 5.70 Digital Video Tracking System.

### Passive Avoidance Test

**Figure 2:**
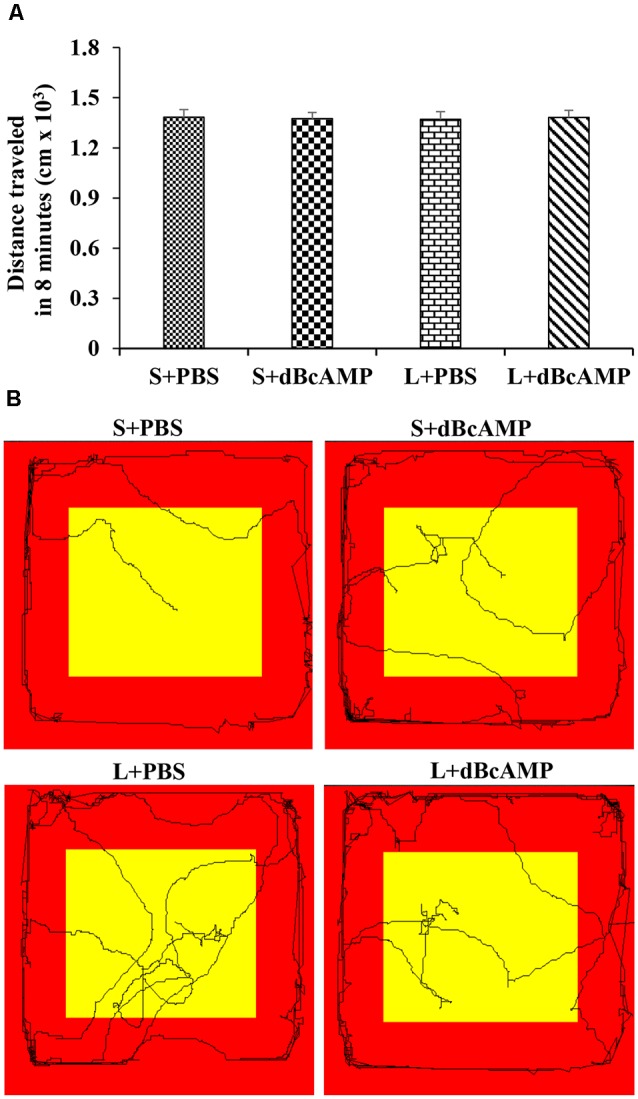
**(A)** Graph showing the distance traveled by mice in the different groups during the screening test before the passive avoidance test. Note that there is no significant difference between any groups (two-way ANOVA, *P* > 0.05; main interaction df-1, *F*_(1,44)_ = 0.05647, *P* = 0.8133; lesion type (L or S) df-1, *F*_(1,44)_ = 0.003144, *P* = 0.9555; treatment type (dBcAMP or PBS) df-1, *F*_(1,44)_ = 0.001009, *P* = 0.9748). **(B)** Video tracking of the mice in the different groups during the screening test.

After the Morris water maze test, the mice (*n* = 12/group) were subjected to a passive avoidance test to test the avoidance learning and memory. We have screened all mice for their locomotor activity in an open-field apparatus prior to the passive avoidance test. The mice were allowed to explore the open-field apparatus [100 cm (L) × 100 cm (B) × 20 cm (H)] for 8 min in each trial, and the distance traveled was measured with an EzVideoTM 5.70 Digital Video Tracking System. For each mouse, three trials were given, and the mean distance traveled in 8 min was calculated. In all groups, only the mice which traveled 1,000–1,500 cm in 8 min were used for the behavioral study (Figure 2). The passive avoidance apparatus was made of Plexiglass. It consisted of a larger bright compartment (40 cm × 40 cm × 40 cm) and a smaller dark compartment (15 cm × 10 cm × 10 cm). The apparatus was kept in a relatively dark room. The bright compartment was illuminated with a 15-W bulb. The dark compartment was dark relative to the bright compartment. Stainless steel rods spaced at equal distance from the floor of the dark compartment were connected to an electrical stimulator (DC current, maximum—500 mA, 10–100 V). The bright and the dark compartments were separated from each other by a sliding door. The apparatus was kept under a video camera of the Ezvideo570DV video tracking system. The mice were trained to explore both the bright and the dark compartments of the passive avoidance apparatus for 5 min. The movement of the mice was tracked by the Ezvideo570DV video tracking system (EzVideo^TM^ 5.70 Digital Video Tracking System). The time spent in the bright and the dark compartments by each mouse was recorded and analyzed by the Ezvideo570DV video tracking system. The trial was repeated three times with 5-min inter-trial interval. At the end of the third trial, the mouse was confined to the dark compartment of the passive avoidance apparatus (by closing the sliding door) and a foot shock from the stimulator (DC, 0.2–0.5 mA) was given for 3 s. At 24 h after the foot shock, the memory retention test was done. During the memory retention test, each mouse was allowed to explore the bright and the dark compartments for 3 min. The time spent in the dark compartment and the dark compartment entry latency were recorded for each mouse.

### Tissue Fixation and Processing for Immunostaining

The mice in all groups (*n* = 6/group) were anesthetized with a ketamine–xylocane cocktail (ketamine 40 mg/kg and xylocaine 5 mg/kg) 1 day after the completion of the behavioral tests. The heart was exposed and perfused with 50 ml of heparinized saline, followed by 200 ml of 4% paraformaldehyde. The brain was dissected and post-fixed for 48 h in the same fixative. The tissues were cryoprotected in 10, 20, and 30% sucrose solution (24 h in each). Then, the tissues were embedded in an optimum cutting temperature compound (Sigma Chemicals, St. Louis, MO, USA). Thirty-micron-thick serial, coronal cryostat sections were taken and stored in phosphate buffer-filled 24-well culture plates. The serial brain sections were stained for Cresyl violet, doublecortin (for neurogenesis), and NeuN (for assessing the neurons).

### Cresyl Violet Staining

The general morphology and the neurodegeneration in the hippocampus were assessed by Cresyl violet staining. The structure of the different regions of the hippocampus was studied. Brain sections mounted on gelatinized slides were air-dried and stained with 0.1% aqueous Cresyl violet stain (Sigma Chemicals, St. Louis, MO, USA) for 20 min at 60°C. Then, the slides were washed in distilled water, differentiated in 70% ethyl alcohol and dehydrated in ascending grades of ethyl alcohol, cleared in xylene, and mounted with DPX (Sigma Chemicals, St. Louis, MO, USA). Observations were done on six serial sections (150 μm apart) from each animal.

### Doublecortin and NeuN Immunostaining and Quantification

Thirty-micron-thick frozen serial, coronal sections were immunostained for doublecortin and NeuN. Endogenous peroxidase activity was reduced by treating the sections with 3% hydrogen peroxide. Nonspecific binding of the antibodies was blocked with 5% normal goat/rabbit serum (Vector Laboratories, Burlingame, CA, USA). The sections were then incubated with goat polyclonal anti-DCX (1:200, Santa Cruz Biotechnology, Dallas, TX, USA) or mouse monoclonal anti-NeuN (1:500, Abcam, Cambridge, MA, USA) antibodies overnight at 4°C. The sections were incubated with biotinylated anti-goat IgG (1:20) or with biotinylated anti-mouse IgG (1:20, Vector Laboratories, Burlingame, CA, USA) for 1 h, followed by incubation with avidin biotin complex (1:20, ABC kit, Vector Laboratories, Burlingame, CA, USA) for another 1 h. The color was developed using diaminobenzidine (DAB) as chromogen for NeuN staining and vector gray for DCX staining. The sections were mounted on gelatin-coated slides, air-dried, dehydrated in ascending grades of ethyl alcohol, cleared in xylene, and coverslipped with DPX. The number of NeuN-positive neurons and the number of DCX-positive newly formed neurons were counted in six randomly selected fields (photographs taken with ×40 objective) in each section from the DG and the CA3 regions in the kainic acid lesion model. From each mouse, six sections (150 μm apart) were selected for quantification. Finally, the number of new neurons (DCX-positive) and the number of mature neurons (NeuN-positive) per cubic millimeter of tissue were calculated for each mouse (Beauquis et al., 2014; Abd-El-Basset and Rao, 2018). Briefly, the total number of DCX-positive neurons and the total number of NeuN-positive neurons per cubic millimeter of tissue were quantified by using the data collected from coronal brain sections using the formula *T* = N*V/*t*, where *N* is the numerical cell density, *V* is the volume of the tissue used for quantification (CA3 region, DG, and dentate hilus), and *t* is section thickness (30 μm). The numerical cell density (*N*) was calculated by measuring the area of the section used for quantification with the NIS-Elements software (NIS-Elements-D2.20) and the total cell counts in six randomly selected fields. The volume (*V*) was calculated by measuring the area of the section used for quantification [CA3, DG (DG+dentate hilus), and dentate hilus region] with the NIS-Elements software (NIS-Elements-D2.20) and multiplying by the section thickness (30 μm), the inter-section distance (60 μm), and the number of sections (6). All of the brain sections were examined with an Olympus BX51 TF upright transmitted light/fluorescence microscope using ×20 (aperture is 0.50) and ×40 (aperture is 0.75) objectives.

### Polyacrylamide Gel Electrophoresis and Immunoblotting

Mice (*n* = 6/group) were perfused with 50 ml of cold saline. The hippocampus was dissected and snap-frozen in liquid nitrogen and stored at −80°C until Western blot analysis for doublecortin. The tissue was thawed and incubated in an ice-cold radioimmunoprecipitation assay lysis buffer [sodium orthovanadate (0.5 mM) and the protease inhibitors, phenylmethanesulfonyl fluoride (1 mM), aprotinin (10 μg/ml), and leupeptin (1 μg/ml)] for 10 min. The tissue was homogenized for 3–5 min at 4°C and centrifuged for 5 min at 14,000 rpm at 4°C. The supernatant was collected for analysis. The protein concentration in the samples was determined using an Epoch^TM^ Multi-volume spectrophotometer system (Biotek, Winooski, VT, USA). The protein standards were made by serial dilution of a concentrated stock of bovine serum albumin (Sigma-Aldrich, Saint Louis, MO, USA, PN-A3294) in double-distilled water. The protein standard curve was made by measuring the absorbance at 280 nm. The protein concentration in the samples was measured by loading 2 μl of the undiluted sample on the Take3^TM^ plate and measuring the absorbance at 280 nm. The proteins in all samples (75 μg protein/well) were resolved on 10% sodium dodecyl sulfate-polyacrylamide gel electrophoresis (SDS-PAGE) gel (Laemmli, 1970). The proteins were transferred to the nitrocellulose membrane (Towbin et al., 1979). After transfer, the membranes were incubated with 5% skim milk in Tris-buffered saline-Tween 20 (TBST) for 1 h. The immunoblots were probed with goat anti-doublecortin antibody (Santa Cruz Biotechnology, Dallas, TX, USA) and rabbit anti-glyceradehde-3-phosphate dehydrogenase (endogenous sample loading control, Sigma Chemicals, St. Louis, MO, USA) diluted in 5% dry milk in TBST. The membranes were then incubated in rabbit anti-goat or goat anti-rabbit secondary antibodies conjugated to horseradish peroxidase (1:1,000; Santa Cruz Biotechnology, Dallas, TX, USA). Enhanced chemiluminescence system (Santa Cruz Biotechnology, Inc., Dallas, TX, USA) was used to visualize the immunoreactive bands. The differences in doublecortin band intensities in the different groups were determined by scanning the bands and quantifying the density in the Image-J image analysis software.

### Statistical Analysis

Data were expressed as mean ± SEM and were analyzed by two-way repeated-measures ANOVA (water maze learning sessions) or two-way ANOVA, followed by Bonferroni’s post-test using GraphPad Prism-8 software. *P*-values < 0.05 were considered as statistically significant.

## Results

### Learning and Memory (Water Maze Test)

**Figure 3:**
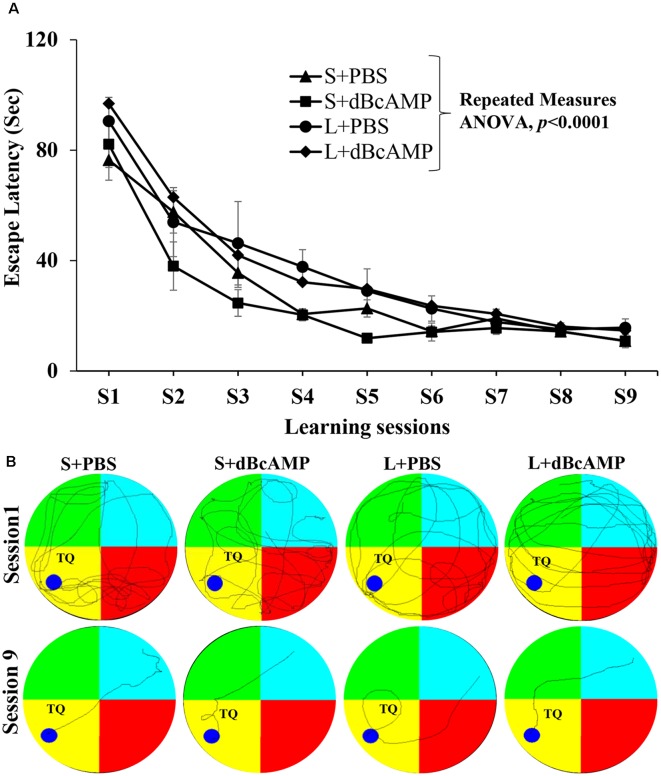
**(A)** Graph showing the escape latency during the water maze learning sessions 1–9. Note that the escape latency gradually decreased from session 1–9 (two-way repeated measures ANOVA, *P* < 0.0001). **(B)** Video tracking of mice in the different groups during the first and ninth learning sessions. A circular platform was submerged in the region, indicated by the dark blue circle, 1 cm below the water level during the learning sessions in the target (platform) quadrant (TQ) of the water maze. Note that the mice in all groups learnt to locate the hidden platform by the ninth session.

Learning as tested by the water maze test showed no significant difference between the groups (Figure 3A). The mice in all groups learned to locate the hidden platform by the ninth session two-way repeated-measures ANOVA—main interaction: df-24, *F*_(24,396)_ = 0.6852, *P* = 0.8670; raw factor (sessions): df-8, *F*_(8,396)_ = 66.71, *P* < 0.0001; column factor (groups): df-3, *F*_(3,396)_ = 8.228, *P* < 0.0001; Bonferroni multiple-comparison test session 1 vs. session 9: *P* < 0.0001 in S+PBS, S+dBcAMP, L+PBS, and L+dBcAMP groups. The inter-group comparison in all sessions is not significantly different (*P* > 0.05) from each other (Figure 3A). The video tracking of mice during the first and ninth learning sessions is shown in Figure 3B.

**Figure 4:**
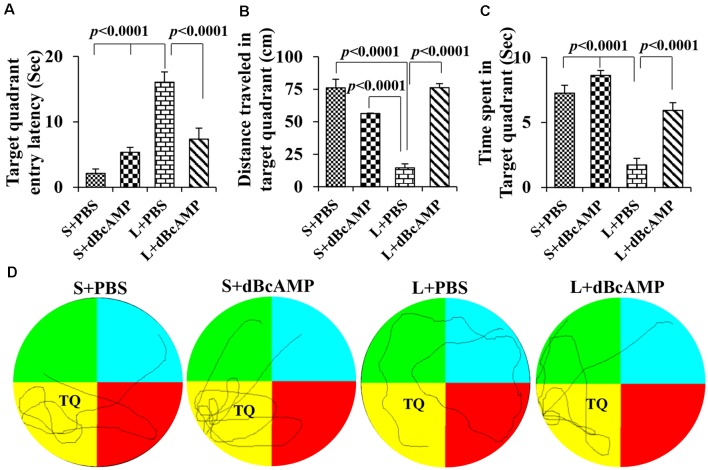
**(A,B,C)** Graphs showing the target quadrant entry latency **(A)**, distance traveled in the target (platform quadrant, **B**), and time spent in the target quadrant **(C)** during the probe test. Note that the target quadrant entry latency was significantly higher in the L+PBS group compared to the S+PBS (*P* < 0.0001) and S+dBcAMP (*P* < 0.0001) groups, and it was decreased significantly in the L+dBcAMP group (*P* < 0.0001). The distance traveled and the time spent in the target quadrant were significantly lower in the L+PBS group compared to those of all other groups (*P* < 0.0001). Two-way ANOVA, Bonferroni’s test, *n* = 12 in all groups. **(D)** Video tracking of mice in the different groups during the probe test (memory retention test) 24 h after the last learning session. The platform was removed from the target/platform quadrant (TQ), where a circular platform was submerged 1 cm below the water level during the learning sessions. Note that S+PBS, S+dBcAMP, and L+dBcAMP mice entered the target quadrant quickly and kept exploring there for the platform. However, mice in L+PBS failed to reach the target quadrant and hence explored the whole maze, suggesting memory impairment.

Memory retention test at 24 h after the last learning session showed a significant memory deficit in the L+PBS mice compared to that in the S+PBS mice. The target quadrant entry latency was significantly increased in the L+PBS group compared to those in the S+PBS (*P* < 0.0001) and the S+dBcAMP (*P* < 0.0001) groups. The target quadrant entry latency was significantly decreased (*P* < 0.001) in the L+dBcAMP group compared to that in the L+PBS group [two-way ANOVA, main interaction—df-1: *F*_(1,44)_ = 22.5, *P* < 0.0001; lesion type (L or S)—df-1: *F*_(1,44)_ = 40.02, *P* < 0.0001; treatment type (dBcAMP or PBS)—df-1, *F*_(1,44)_ = 4.77, *P* = 0.0343, Figure 4A]. The distance traveled and the time spent in the target quadrant were significantly decreased in the L+PBS group compared to those in the S+PBS (*P* < 0.0001) and the S+dBcAMP (*P* < 0.0001) groups. The distance traveled and the time spent in the target quadrant were significantly increased in the L+dBcAMP group compared to those in the L+PBS group [(*P* < 0.0001, Figures 4B,C; distance traveled: main interaction—df-1, *F*_(1,44)_ = 103.2, *P* < 0.0001; lesion type (L or S)—df-1, *F*_(1,44)_ = 26.96, *P* < 0.0001; treatment type (dBcAMP or PBS)—df-1, *F*_(1,44)_ = 27.36, *P* < 0.0001; time spent: main interaction—df-1, *F*_(1,44)_ = 7.205, *P* = 0.0102; lesion type (L or S)—df-1, *F*_(1,44)_ = 60.85, *P* < 0.0001; treatment type (dBcAMP or PBS)—df-1, *F*_(1,44)_ = 27.82, *P* < 0.0001)]. The video tracking of mice during the memory retention test is shown in Figure 4D.

### Passive Avoidance Test

**Figure 5:**
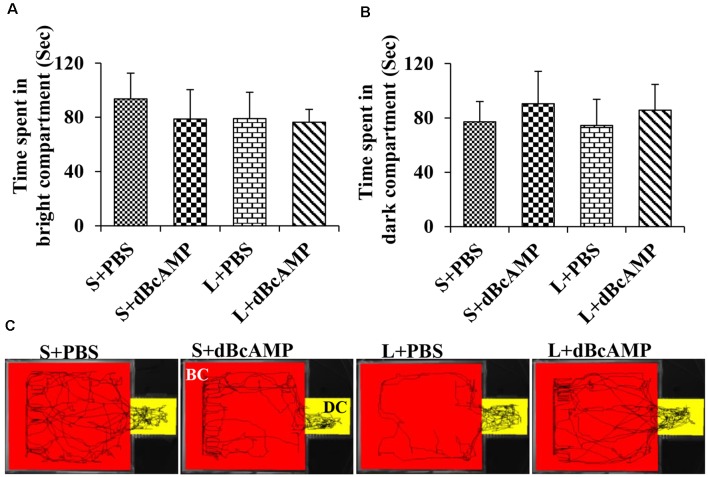
**(A,B)** Graph showing the time spent in the bright and in the dark compartments of the passive avoidance apparatus by mice in the different groups during the passive avoidance learning session. Note that there is no significant difference between any groups. **(C)** Video tracking of mice in the different groups during the last passive avoidance learning session. BC, bright compartment; DC, dark compartments of the passive avoidance apparatus. Note that the mice in all groups explored both the bright and the dark compartments.

**Figure 6:**
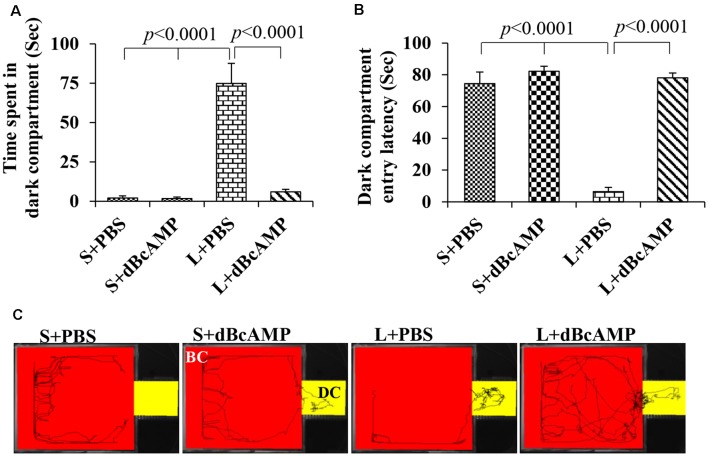
**(A,B)** Graph showing the time spent in the dark compartment and dark compartment entry latency in the different groups during the passive avoidance memory retention test. Note that L+PBS mice spent significantly more time in the dark compartment compared to mice in the S+PBS and the S+dBcAMP groups (*P* < 0.0001). However, L+dBcAMP mice spent significantly less time in the dark compartment compared to L+PBS mice (*P* < 0.0001), like the S+PBS and the S+dBcAMP groups, suggesting significant memory retention. Similarly, dark compartment entry latency was significantly less in L+PBS compared to S+PBS and S+dBcAMP (*P* < 0.0001), and it is increased significantly in the L+dBcAMP group. **(C)** Video tracking of mice in the different groups during the passive avoidance memory retention test 24 h after the last learning session. Note that mice in L+PBS entered the dark compartment (DC) as soon as the test started, suggesting poor memory retention. However, mice in S+PBS, S+dBcAMP, and L+dBcAMP explored only the bright compartments (BC) except for one entry to the dark compartment (DC).

The passive avoidance learning session showed no significant difference (*P* > 0.05) in the time spent in the bright or the dark compartment between the groups [bright compartment: main interaction—df-1, *F*_(1,44)_ = 0.1149, *P* = 0.7362; lesion type (L or S)—df-1, *F*_(1,44)_ = 0.2208, *P* = 0.6407; treatment type (dBcAMP or PBS)—df-1, *F*_(1,44)_ = 0.2342, *P* = 0.6308); dark compartment: main interaction—df-1, *F*_(1,44)_ = 0.003214, *P* = 0.9550; lesion type (L or S)—df-1, *F*_(1,44)_ = 0.03687, *P* = 0.8486, treatment type (dBcAMP or PBS)—df-1, *F*_(1,44)_ = 0.3926, *P* = 0.5342)]. The mice in all groups explored both the dark and the bright compartments (Figures 5A,B). The video tracking of mice during the learning sessions is shown in Figure 5C. Memory retention test at 24 h after the last learning session showed a significant memory deficit in the L+PBS mice compared to that in the S+PBS mice. The time spent in the dark compartment was significantly more in the L+PBS group compared to those in the S+PBS (*P* < 0.0001) and the S+dBcAMP groups (*P* < 0.0001, Figure 6A). The L+dBcAMP group spent significantly less time in the dark compartment compared to the L+PBS group [main interaction—df-1, *F*_(1,44)_ = 28.09, *P* < 0.0001; lesion type (L or S)—df-1, *F*_(1,44)_ = 35.47, *P* < 0.0001; treatment type (dBcAMP or PBS)—df-1, *F*_(1,44)_ = 28.68, *P* < 0.0001]. The dark compartment entry latency was significantly more in the S+PBS and the S+dBcAMP groups compared to that in L+PBS (*P* < 0.0001, Figure 6B). The dark compartment entry latency was significantly more in the L+dBcAMP group compared to that in L+PBS, indicating a better avoidance memory [main interaction—df-1, *F*_(1,44)_ = 19.22, *P* < 0.0001; lesion type (L or S)—df-1, *F*_(1,44)_ = 24.64, *P* < 0.0001; treatment type (dBcAMP or PBS)—df-1, *F*_(1,44)_ = 29.85, *P* < 0.0001, Figure 6B]. The video tracking of mice during the memory retention test is shown in Figure 6C.

### Morphological Changes in the Hippocampus

#### Cresyl Violet Staining

**Figure 7:**
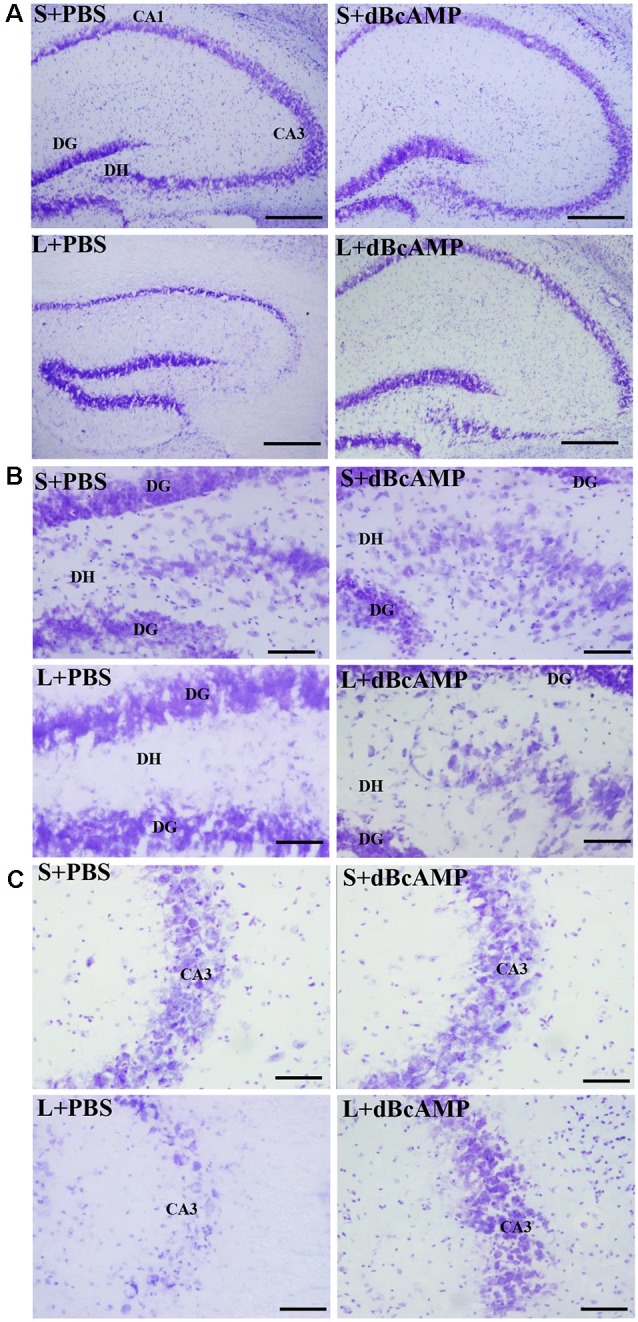
Photomicrographs of the hippocampus **(A)**, dentate gyrus (DG)—hilus region **(B)**, and CA3 region **(C)** stained with Cresyl violet to analyze the hippocampal morphology in the different groups. Note the significant degenerative changes in the CA3 and the dentate hilus (DH) regions in the L+PBS group compared to the S+PBS and the S+dBcAMP groups. Treatment with dBcAMP (L+dBcAMP) decreased the degenerative changes and protected the neurons from degeneration both in the CA3 and the DH regions compared to the L+PBS group. Scale bar = 100 μm in **(A)**, scale bar = 10 μm in **(B,C)**.

Cresyl violet staining of the hippocampal sections showed decreased neurons in the CA3 and the dentate hilus (DH) regions in the L+PBS group compared to those in the S+PBS and the S+dBcAMP groups. Treatment with dBcAMP (L+dBcAMP) protected the neurons from degeneration both in the CA3 and the DH regions compared to that in the L+PBS group (Figures 7A–C).

#### NeuN Immunostaining

**Figure 8:**
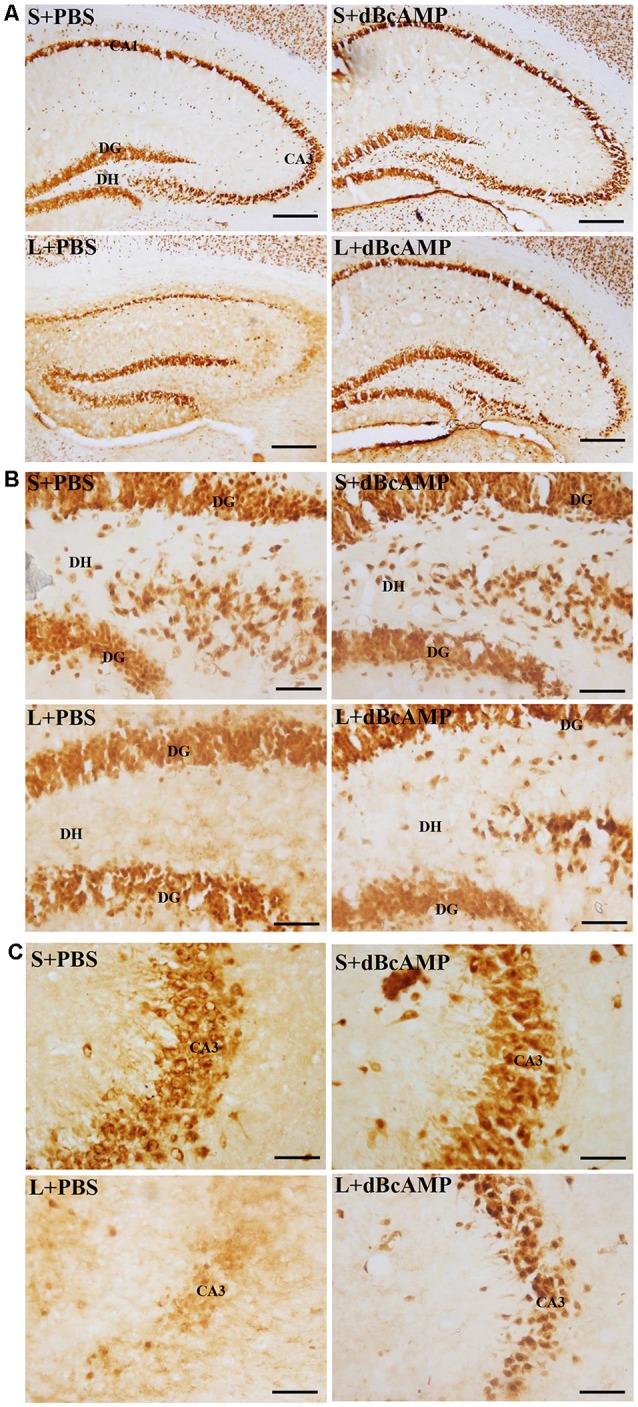
Photomicrographs of the hippocampus **(A)**, DG–hilus region **(B)**, and CA3 region **(C)** immunostained for NeuN to analyze the number of neurons in the CA3 and in the dentate hilus regions. Note the decreased number of neurons in the CA3 and in the dentate hilus (DH) regions in the L+PBS group compared to the S+PBS and the S+dBcAMP groups. Treatment with dBcAMP (L+dBcAMP) protected the neurons from degeneration, both in the CA3 and in the DH region compared to the L+PBS group. Scale bar = 100 μm in **(A)**, scale bar = 10 μm in **(B,C)**.

**Figure 9:**
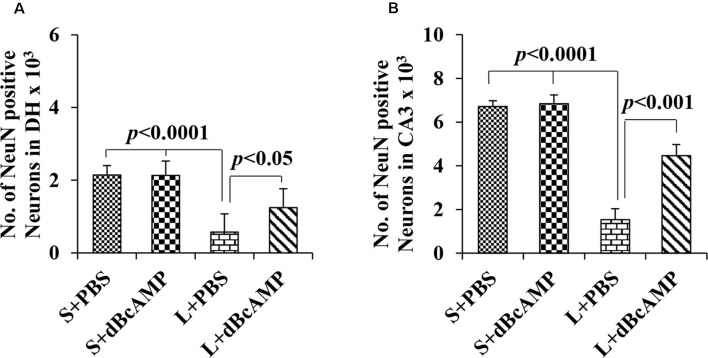
Graph showing the number of NeuN-positive neurons in the dentate hilus (DH; **A**) and in the CA3 region **(B)**. Note the significantly decreased number of neurons in the L+PBS group compared to the S+PBS and the L+dBcAMP groups (*P* < 0.0001) in both the DH and the CA3 regions. However, the number of neurons increased significantly in the L+dBcAMP group compared to that in the L+PBS group (*P* < 0.05). Two-way ANOVA, Bonferroni’s test, *n* = 6 in all groups.

NeuN immunostaining of the hippocampal section confirmed the decreased neurons in the DH and the CA3 regions in the L+PBS group compared to those in the S+PBS and the S+dBcAMP groups. Further, treatment with dBcAMP (L+dBcAMP) protected the neurons from degeneration both in the DH and the CA3 regions compared to that in the L+PBS group (Figures 8A–C). Quantification of neurons in the dentate hilus and the CA3 regions showed significantly decreased neurons in the dentate hilus (*P* < 0.0001) and CA3 regions (*P* < 0.0001) in the L+PBS group compared to those in the S+PBS and the S+dBcAMP groups (Figures 9A,B). Treatment with dBcAMP (L+dBcAMP) significantly increased the number of neurons in both the DH (*P* < 0.05) and the CA3 (*P* < 0.001) regions compared to that in the L+PBS group (DH: main interaction—df-1, *F*_(1,20)_ = 4.545, *P* = 0.0456; lesion type (L or S)—df-1, *F*_(1,20)_ = 48.3, *P* < 0.0001; treatment type (dBcAMP or PBS)—df-1, *F*_(1,20)_ = 4.211, *P* = 0.0535; CA3: main interaction—df-1, *F*_(1,20)_ = 10.55, *P* = 0.0040; lesion type (L or S)—df-1, *F*_(1,20)_ = 77.35, *P* < 0.0001; treatment type (dBcAMP or PBS)—df-1, *F*_(1,20)_ = 12.55, *P* = 0.0020, Figures 9A,B).

#### Hippocampal Neurogenesis

**Figure 10:**
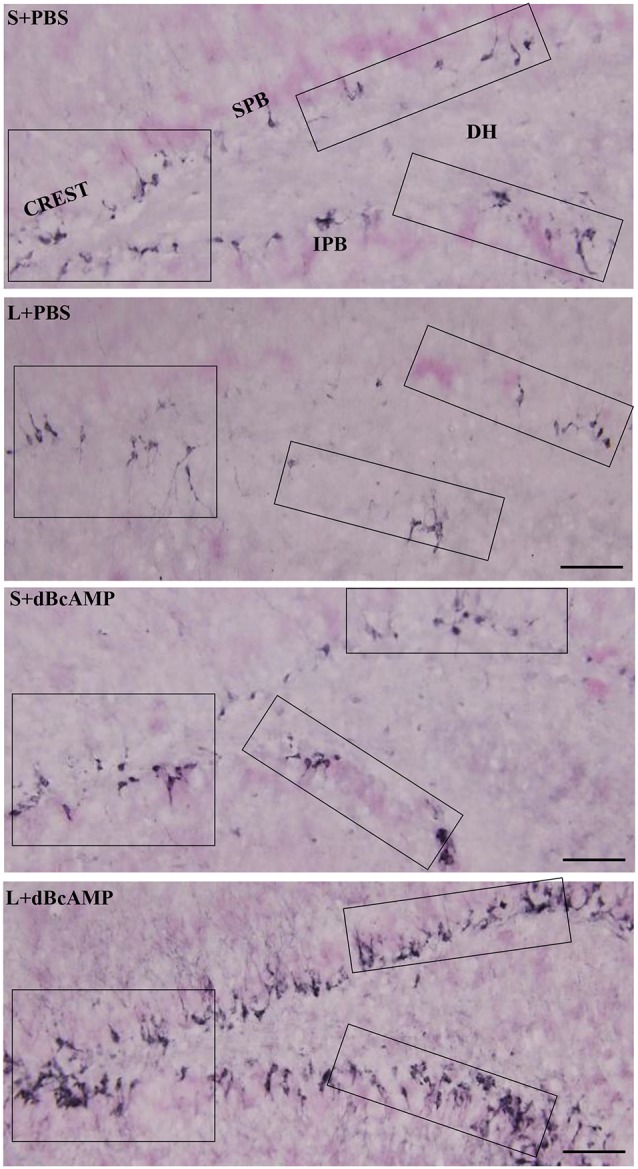
Photomicrographs of the entire hippocampal DG immunostained for doublecortin (DCX) to analyze the neurogenesis. Crest (CREST), suprapyramidal blade (SPB), and infrapyramidal blade (IPB) regions indicated in boxes in all groups are shown at higher magnification in Figures 11A–C. Note the noticeable decrease in DCX-positive neurons in the L+PBS group compared to those in the S+PBS and the S+dBcAMP groups in all regions. Treatment with dBcAMP (L+dBcAMP) enhanced the hippocampal neurogenesis in all regions compared to that in the L+PBS group. DH, dentate hilus. Scale bar = 20 μm.

**Figure 11:**
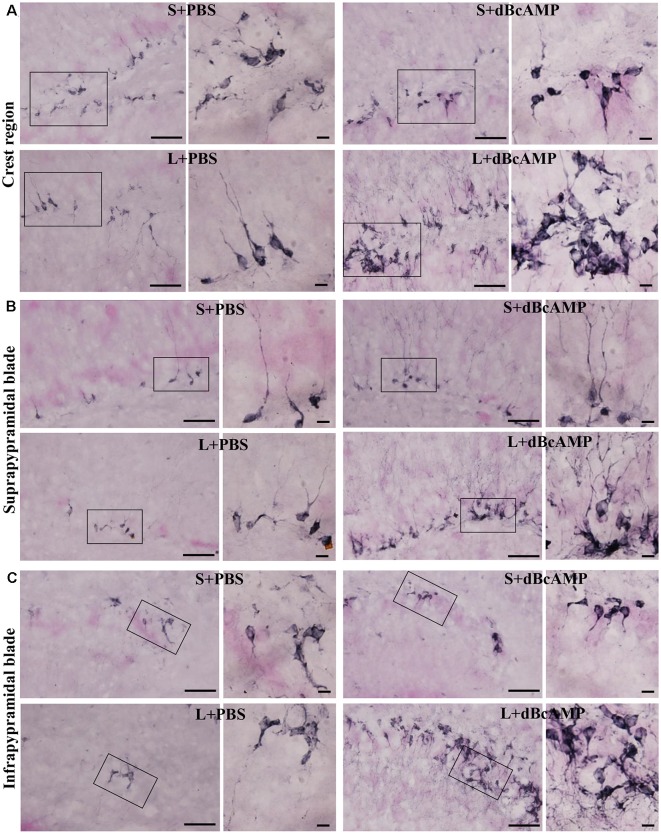
Photomicrographs of the hippocampal DG immunostained for doublecortin (DCX) to analyze the neurogenesis (regions indicated by boxes in Figure 10 are shown at higher magnification). **(A)** Crest (CREST), **(B)** suprapyramidal blade (SPB), and **(C)** infrapyramidal blade (IPB) regions. Selected regions (rectangular box) of CREST, SPB, and IPB are shown at higher magnification in the adjacent photomicrograph. Note the noticeably decreased DCX-positive neurons in the L+PBS group compared to those in the S+PBS and the S+dBcAMP groups in all regions. Treatment with dBcAMP (L+dBcAMP) enhanced the hippocampal neurogenesis in all regions compared to that in the L+PBS group. Scale bar = 10 μm. DH, dentate hilus.

**Figure 12:**
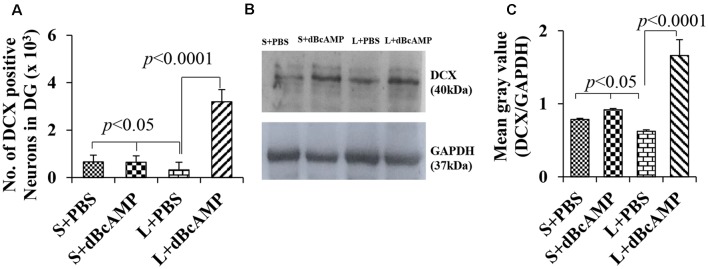
**(A)** Graph showing the number of doublecortin (DCX)-positive neurons in the entire DG. **(B)** DCX immunoblot. **(C)** Mean gray value (DCX/glyceradehde-3-phosphate dehydrogenase). Note the significantly decreased number of new neurons **(A)** and DCX content **(B,C)** in the L+PBS group compared to those in the S+PBS and the L+dBcAMP groups (*P* < 0.05). However, the number of neurons and the DCX content increased significantly in the L+dBcAMP group compared to those in the L+PBS group (*P* < 0.0001). Two-way ANOVA, Bonferroni’s test, *n* = 6 in all groups.

Doublecortin immunostaining showed a markedly decreased generation of new neurons in the hippocampal DG of the L+PBS group compared to those of the S+PBS and the S+dBcAMP groups; treatment with dBcAMP enhanced the generation of the new neurons in L+dBcAMP (Figures 10, 11A–C). The quantification of new neurons in the hippocampal DG revealed a significant decrease in the generation of new neurons in the L+PBS group compared to those in the S+PBS and the S+dBcAMP groups (*P* < 0.05, Figure 10). Treatment with dBcAMP (L+ dBcAMP) significantly (*P* < 0.0001) increased the number of new neurons in the hippocampal DG compared to that in the L+PBS group [main interaction—df-1, *F*_(1,20)_ = 69.46, *P* < 0.0001; lesion type (L or S)—df-1, *F*_(1,20)_ = 16.9, *P* = 0.0005; treatment type (dBcAMP or PBS)—df-1, *F*_(1,20)_ = 87.97, *P* < 0.0001, Figure 12A].

### Western Blot Analysis of Doublecortin

Western blot analysis for doublecortin confirmed the markedly decreased generation of new neurons in the hippocampus of the L+PBS group compared to those in the S+PBS and the S+dBcAMP groups and the enhanced hippocampal neurogenesis in the dBcAMP-treated group (Figure 10). The quantification of the immunoblot revealed a significant decrease in DCX content in the L+PBS group compared to those in the S+PBS and the S+dBcAMP groups (*P* < 0.05, Figure 12B). Treatment with dBcAMP (L+dBcAMP) significantly (*P* < 0.0001) increased the DCX content compared to that in the L+PBS group [main interaction—df-1, *F*_(1,20)_ = 52.53, *P* < 0.0001; lesion type (L or S)—df-1, *F*_(1,20)_ = 21.63, *P* = 0.0002; treatment type (dBcAMP or PBS)—df-1, *F*_(1,20)_ = 87.75, *P* < 0.0001, Figure 12C].

In summary, in the water maze learning sessions, there was no significant difference in escape latency between the groups, suggesting the unimpaired learning ability of mice in all groups. The memory retention test showed that the L+dBcAMP mice had a significantly short entry latency and higher target quadrant time/distance traveled compared to the L+PBS group, suggesting better memory retention.

The passive avoidance test showed that the L+dBcAMP mice had a significantly improved memory retention compared to the L+PBS mice. The morphological studies showed a significantly more number of neurons in the dentate hilus and the CA3 regions and enhanced hippocampal neurogenesis in the L+dBcAMP compared to that in the L+PBS group. There was no significant difference between the S+dBcAMP and the S+PBS groups in the water maze/passive avoidance tests and the number of newly born neurons.

## Discussion

### Excitotoxic Lesion (ICV-KA Model)

In the present study, we used a kainic acid-induced excitotoxic injury model. The injection of kainic acid (0.25 μg/ventricle, bilaterally) produced only a few (two to four) events of status epileptics during the 3-h post-surgery period. The status epileptics lasted for a few minutes only. However, we have not addressed the electrographic seizures in the ICV-KA-injected and the dBcAMP- and PBS-treated mice. The mice which underwent sham surgery did not show any epilepsy. Both the excitotoxically injured—injured and treated with dBcAMP—and sham surgery mice were healthy during the post-surgical period. Epileptiform encephalographic (EEG) events, which are associated with limbic-type behavioral seizures, have been reported in rats subjected to microdialysis with 10 mM dBcAMP intrahippocampally (Ludvig et al., 1992). However, in our study, the mice with intracerebro-ventricular injection of kainic acid at 0.25 μg/ventricle and treated with dBcAMP did not show any behavioral seizures, even though a significant lesion in the hippocampus was produced. However, it might have produced epileptiform EEG in the dBcAMP-treated mice during the early post-injury period, which we have not studied.

### dBcAMP and Learning and Memory

Memory retention test as assessed by the Morris water maze test and the passive avoidance test revealed significant memory impairment in the hippocampus-injured mice, and memory impairment was decreased by treatment with dBcAMP. Screening of mice for their swimming ability and locomotor activity before subjecting them to the behavioral tests did not show any swimming deficit or locomotor deficit among them. Hence, locomotor activity may not have contributed to the observed behavioral deficit. Our results were consistent with those of other studies. Sharifzadeh et al. (2007) showed the preventive role of dBcAMP in spatial memory impairment in the Morris water maze test by increasing the cAMP level. dBcAMP, a synthetic analog of cAMP, is highly permeable through the cell membrane and it mimics the activity of endogenous cAMP (Hosseini-Zare et al., 2011). dBcAMP is also reported to improve auditory/audio memory (Nassireslami et al., 2013). dBcAMP is reported to simulate the distinct components of long-term potentiation (population spike and population excitatory post-synaptic potential slope) in the CA1 region of rat hippocampus in culture (Slack and Walsh, 1995). A recent study showed that memory impairment was induced by beta-amyloid protein, and treatment with dBcAMP prevented such spatial memory impairment as evaluated by the Morris water maze test (Aghsami et al., 2018).

### Neuroprotection by dBcAMP—Role of Astrocytes and Microglia

In the present study, we observed a significant neuroprotection in the hippocampal CA3 and dentate hilus regions in kainic acid-injured mice that were treated with dBcAMP. This neuroprotection may be mediated by astrocytes and microglia in the vicinity of the injury. Microglial activation after injury leads to the release of a variety of pro-inflammatory cytokines and free radicals. These cytokines and free radicals induce the degenerative process (Kim and de Vellis, 2005; Block et al., 2007). In gliosis, the impaired handling of extracellular glutamate by astrocytes in the regions of gliosis leads to excitotoxic neuronal damages. The microglia are recognized as a potent inducer of astrocyte activation and proinflammatory mediators. Tilleux et al. (2009) reported the enhanced expression of GLT-1 in astrocytes that are treated with dBcAMP or astrocytes that are exposed to soluble mediators released by the activated microglia. Matsuura et al. (2002) demonstrated a markedly increased glutamate transport activity, in a concentration- and time-dependent manner, in astrocytes in cultures that are treated with dBcAMP. Thus, the neuroprotection by dBcAMP may be attributed to enhanced gliosis and hence decreased the excitotoxicity and neuronal death. Indeed we have reported earlier enhanced astrocytes and microglial population in the CA3 and the DG regions of the hippocampus within a week after the excitotoxic lesion was induced by an intracerebro-ventricular injection of kainic acid (Abd-El-Basset and Rao, 2018).

### dBcAMP and Neurogenesis

An analysis of hippocampal neurogenesis by labeling the newly formed neurons with anti-doublecortin antibody showed a significant decrease in hippocampal neurogenesis in the kainic acid-injured hippocampus at 4 weeks after the injury. However, hippocampal neurogenesis was found to be enhanced in those injured mice treated with dBcAMP. In our study, we have killed the mice on the 36th day of the experiment (i.e., 29 days after the last dose of dBcAMP or PBS treatment) and quantified the number of doublecortin-positive neurons in the hippocampal DG. In this study, we have not addressed the survival and the maturation of newly generated neurons. Several studies have shown the transition of newborn neurons into mature neurons (Rao and Shetty, 2004; Rao et al., 2005). We used DCX as a marker for new neurons in the DG. This is based on the fact that new neurons that are predominantly born during the 12 days prior to euthanasia can be visualized with DCX immunostaining (Rao and Shetty, 2004). Evidence from both *in vivo* and *in vitro* studies suggests that hippocampal neurogenesis is linked to an increase in the intracellular cAMP, a second messenger that regulates many functions, including metabolism, cell proliferation, and neuronal signaling, by altering gene expression (West et al., 2001; Nicot et al., 2002; Fujioka et al., 2004; Gabellini, 2004; Pandey, 2004). The cAMP cascade in the neuronal cells can be triggered by the same conditions known to influence hippocampal neurogenesis, such as neural injury (Nakagawa et al., 2002). Thus, in our study, the initial lesion by kainic acid itself might have triggered the cAMP cascade in the neural progenitor cells in the DG; further, dBcAMP injected for 7 days enhanced the intracellular cAMP, resulting in enhanced hippocampal neurogenesis.

Adenylyl cyclase (a potent stimulator of adenylyl cyclase) regulates neurogenesis in prenatal hindbrain and cortical neuroblasts (Lu and DiCicco-Bloom, 1997; Waschek et al., 1998; Suh et al., 2001). A recent study showed that Lot1 (a zinc finger transcription factor) expression in CGC is cAMP dependent as treatments with adenylyl cyclase activator increased Lot1 expression at both the mRNA and the protein levels, and dibutyryl cAMP mimicked the actions of Lot1 expression (Contestabile et al., 2005). In addition, dBcAMP is reported to increase the recruitment of neural stem cells into the brain parenchyma and the olfactory bulb in experimental autoimmune encephalomyelitis mice (Khezri et al., 2013). These evidences further support our finding that treatment with dBcAMP has enhanced the DG neurogenesis.

### dBcAMP, Growth Factors, and Neurogenesis

Literature shows a close relation between dBcAMP, growth factors, and neurogenesis. dBcAMP and growth factors have several effects on neurons *in vitro* and *in vivo*. dBcAMP facilitates Neuro-2A (a mouse neural crest-derived cell line) cell differentiation into dopaminergic neurons through the cyclic AMP-responsive element-binding protein (Tremblay et al., 2010). The co-treatment of nerve growth factor (NGF) and dBcAMP exhibits a synergistic effect on neurite outgrowth in PC12 cells in culture (Ng et al., 2009). The VEGF, which regulates angiogenesis, also induces neurogenesis *in vitro* as well as *in vivo* (Sondell et al., 1999; Yancopoulos et al., 2000; Jin et al., 2002). The BDNF and dbcAMP are shown to decrease intracellular free radicals, reactive oxygen species, O^2−^, nitrite, glutathione, and catalase in cultures exposed to ethanol or ethanol-activated microglial-conditioned medium (Boyadjieva and Sarkar, 2013). BDNF, glial-derived growth factor (GDNF), and dBcAMP produce convergent signals to activate protein kinase-A and mitogen-activated protein kinase pathways which are involved in the survival of post-natal mesencephalic dopaminergic neurons *in vitro* (Lara et al., 2003). The survival and the growth of dopaminergic neurons *in vitro* was shown to be increased significantly by dBcAMP and enhanced when it is used along with GDNF or BDNF (Lara et al., 2003). The neurotrophic factors (BDNF, bFGF, VEGF, NGF, and GDNF) are known to stimulate adult neurogenesis (Cao et al., 2004; Kang and Hébert, 2015; Wei et al., 2015; Han et al., 2017; Numakawa et al., 2018). Thus, the observed enhanced neurogenesis in our present study may be due to the enhancement of the abovementioned neurotrophic factor/s. These neurotrophic factors might have stimulated the stem cells in the SGZ of the DG and facilitated their differentiation into neurons, their long-term survival, and their integration into the adult DG and contributed to functional recovery. Indeed in our earlier study we showed neuroprotection by dBcAMP in brain injury models through enhancing the production of BDNF (Abd-El-Basset and Rao, 2018).

## Conclusion

dBcAMP protects the hippocampal neuron from degeneration and enhances neurogenesis, thereby enhancing learning and memory.

## Data Availability Statement

The raw data supporting the conclusions of this article will be made available by the authors, without undue reservation, to any qualified researcher.

## Ethics Statement

The protocol was reviewed and approved by Animal ethical committee of Faculty of Medicine, Health Sciences Center, Kuwait University, Kuwait, 13110 and carried out in accordance with recommendations of NIH Guidelines and Guide for the Care and Use of Laboratory animals.

## Author Contributions

EA and MR contributed for the design of the experiment, data analysis, and manuscript writing.

## Conflict of Interest

The authors declare that the research was conducted in the absence of any commercial or financial relationships that could be construed as a potential conflict of interest.
